# Evidence of MexT-Independent Overexpression of MexEF-OprN Multidrug Efflux Pump of *Pseudomonas aeruginosa* in Presence of Metabolic Stress

**DOI:** 10.1371/journal.pone.0026520

**Published:** 2011-10-24

**Authors:** Ayush Kumar, Herbert P. Schweizer

**Affiliations:** 1 Antimicrobial Resistance Research Group (ARRG), Applied Bioscience Program, Faculty of Health Sciences, University of Ontario Institute of Technology, Oshawa, Ontario, Canada; 2 Department of Microbiology, Immunology and Pathology, Rocky Mountain Regional Center of Excellence for Biodefense and Emerging Infectious Diseases Research, Colorado State University, Fort Collins, Colorado, United States of America; Universite Libre de Bruxelles, Belgium

## Abstract

**Background:**

The *Pseudomonas aeruginosa* MexEF-OprN efflux pump confers resistance to clinically significant antibiotics. Regulation of *mexEF-oprN* operon expression is multifaceted with the MexT activator being one of the most prominent regulatory proteins.

**Methodology:**

We have exploited the impaired metabolic fitness of a *P. aeruginosa* mutant strain lacking several efflux pump of the resistance nodulation cell division superfamily and the TolC homolog OpmH, and isolated derivatives (large colony variants) that regained fitness by incubation on nutrient-rich medium in the absence of antibiotics. Although the *mexEF-oprN* operon is uninducible in this mutant due to a 8-bp *mexT* insertion present in some *P. aeruginosa* PAO1 strains, the large colony variants expressed high levels of MexEF-OprN. Unlike large colony variants obtained after plating on antibiotic containing medium which expressed *mexEF-oprN* in a MexT-dependent fashion as evidenced by clean excision of the 8-bp insertion from *mexT*, *mexEF-oprN* expression was MexT-independent in the large colony variants obtained by plating on LB alone since the *mexT* gene remained inactivated. A search for possible regulators of *mexEF-oprN* expression using transposon mutagenesis and genomic library expression approaches yielded several candidates but proved inconclusive.

**Significance:**

Our results show that antibiotic and metabolic stress lead to up-regulation of MexEF-OprN expression via different mechanisms and that MexEF-OprN does not only extrude antimicrobials but rather serves other important metabolic functions.

## Introduction

*Pseudomonas aeruginosa* is a non-fermentative Gram-negative nosocomial pathogen of significant clinical relevance. It is known to cause a variety of infections including pneumonia, bloodstream infections, urinary tract infections, endocarditis, and burn wound infections [1], [2], [3], [4], [5], [6], [7]. Infections caused by *P. aeruginosa* pose a considerable challenge in the clinical settings owing to its high intrinsic resistance to almost all antibiotics in clinical use [8], [9], [10], [11], [12], [13], [14]. Energy-mediated efflux of antibiotics by Resistance-Nodulation-Cell Division (RND) pumps in *P. aeruginosa* is considered the major factor responsible for its high antibiotic resistance. To date, 11 different RND pumps, capable of effluxing antibiotics/antimicrobial products, have been characterized in *P. aeruginosa* that include MexAB-OprM [15], MexCD-OprJ [16], MexEF-OprN [17], MexGHI-OpmD [18], MexJK-OprM/OpmH [19], MexMN [20], MexPQ-OpmE [20], MexVW-OprM [21], MexXY-OprM [22], TriABC-OpmH [23], and MuxABC-OpmB [24]. Expression of these pumps is usually under tight regulatory control, however molecular mechanisms that regulate the expression of a number of these pumps are not fully understood. Elucidating the mechanisms of regulation of RND pump will not only aid in a better understanding of the antibiotic resistance of *P. aeruginosa* but will also provide valuable insights into their natural function.

The MexEF-OprN pump is the only positively regulated RND pump of *P. aeruginosa* and its expression is activated by a LysR family protein, MexT, encoded by a gene located upstream of the *mexEF-oprN* operon [25]. It has been shown to efflux fluoroquinolones, trimethoprim, and chloramphenicol [17], [26]. MexEF-OprN overexpression has also been shown to be associated with a concurrent downregulation of the outermembrane protein OprD, which in turn results in decreased susceptibility to imipenem [17], [27]. Interestingly, a number of isolates of *P. aeruginosa* have been reported that contain a 8-bp insertion in the *mexT* gene that results in an inactive MexT protein that is unable to activate the expression of *mexEF-oprN* operon [28]. In addition, the regulation of *mexEF-oprN* operon has been shown to be controlled by MexS, an oxidoreductase, as well [27]. MvaT, a global regulator of virulence genes in *P. aeruginosa*, has also shown to be able to repress the expression of the MexEF-OprN pump [29]. In addition to resistance to antibiotics, the overexpression of the MexEF-OprN pump was linked to reduced production of extracellular virulence factors like pyocyanin, elastase, and rhamnolipids [30]. Recent studies also link MexT with the expression of the type III secretion system in *P. aeruginosa* [31]. MexEF-OprN overexpression has also been observed in response to nitrosative stress in absence of any antibiotics [32], [33]. These studies indicate involvement of a complex *mexEF-oprN* regulatory network which largely remains to be elucidated.

The present study was performed to advance our understanding of the regulatory mechanisms involved in the expression of MexEF-OprN. We show that, in the absence of other RND pumps, the expression of MexEF-OprN pump can be activated in a nutrient rich-medium in absence of an antibiotic stress independent of the MexT protein. We also identify putative novel proteins that could be involved in the regulation of this RND pump.

## Results

### A *P. aeruginosa* multiple efflux pump-deficient mutant regains fitness by overexpressing MexEF-OprN

**Figure 1:**
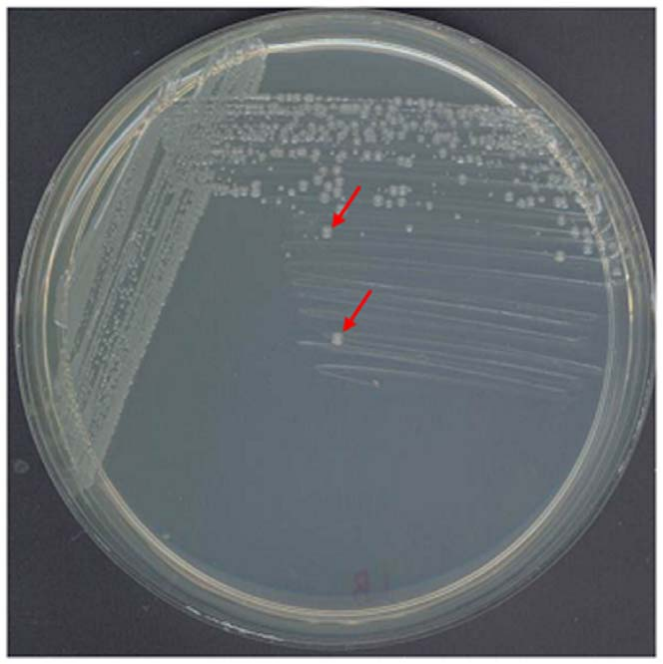
Emergence of large colony variants expressing *mexEF-oprN*. *P. aeruginosa* PAO386 was plated on LB agar and incubated at room temperature for 5 d, following an initial overnight incubation at 37°C. Large colony variants emerged at a high frequency (examples are indicated by red arrows). One such variant (named PAO573) was isolated and retained for further examination.

*P. aeruginosa* PAO386 (Table 1) was used for our studies. This strain contains deletions in four different RND pump encoding operons, namely *mexAB-oprM*, *mexCD-oprJ*, *mexJK*, and *mexXY*. In addition, it also lacks the structural gene for the outer membrane protein OpmH, the *P. aeruginosa* TolC homolog. The MexEF-OprN pump is not expressed in PAO386 as a result of an 8-bp insertion in the *mexT* gene. This strain exhibits a slow growing phenotype and the colonies on LB agar (without supplementation of any antibiotics) have a pin-point morphology after an overnight incubation at 37°C. However, following an extended incubation (5–7 days) at room temperature, a few large-colony variants (with stable phenotype, for example unsectored) were observed (Fig. 1). One such colony was isolated, designated PAO573, and retained for further characterization.

**Table 1:** *P. aeruginosa* strains used in this study.

Strain	Relevant characteristics	Source/Reference
PAO1	*P. aeruginosa* prototroph. Contains the 8-bp insertion in the *mexT* gene, and thus the *mexEF-oprN* operon is uninducible.	[46]
PAO327	PAO1 with Δ(*mexAB-oprM*) Δ(*mexCD-oprJ*) Δ(*mexXY*)	[19]
PAO386	PAO1 with Δ(*mexAB-oprM*) Δ(*mexCD-oprJ*) Δ(*mexJK*) Δ(*mexXY*) Δ*opmH*.	This study
PAO393	Cp^r^ mutant expressing *mexEF-oprN* derived from PAO386 plated on Cp-supplemented LB agar	This study
PAO573	Large colony variant expressing *mexEF-oprN* derived from PAO386 plated on LB agar	This study
PAO599	Gm^r^; PAO386::mini-Tn*7*T-Gm-*mexE_p_-lacZ*	This study
PAO600	Gm^r^; PAO393::mini-Tn*7*T-Gm-*mexE_p_-lacZ*	This study
PAO601	Gm^r^; PAO573::mini-Tn*7*T-Gm-*mexE_p_-lacZ*	This study
PAO602	Gm^r^, PAO1::mini-Tn*7*T-Gm-*mexE_p_-lacZ*	This study
PAO604	PAO1::mini-Tn*7*T-*mexE_p_-lacZ*; Gm-cassette deleted from PAO602	This study
PAO605	PAO386::mini-Tn*7*T-*mexE_p_-lacZ*; Gm-cassette deleted from PAO599.	This study
PAO606	PAO393::mini-Tn*7*T-*mexE_p_-lacZ*; Gm-cassette deleted from PAO600	This study
PAO607	PAO573::mini-Tn*7*T-*mexE_p_-lacZ*; Gm-cassette deleted from PAO601	This study
PAO706	Gm^r^; PAO327::mini-Tn*7*T-Gm-*mexE_p_-lacZ*	This study
PAO707	PAO327::mini-Tn*7*T-*mexE_p_-lacZ*; Gm-cassette deleted from PAO706	This study
PAO709	Gm^r^; PAO707 with Δ*PA02050*::Gm	This study
PAO712	Gm^r^; PAO707 with Δ*PA0487*::Gm	This study
PAO719	PAO707 with Δ*PA0487*; Gm-cassette deleted from PAO712	This study
8485	Tc^r^; *mvaT*::IS*lacZ*/hah (transposon insertion at nucleotide 57 relative to the start codon)	UWGC
PAO1081	Gm^r^; 8485::mini-Tn*7*T-Gm-*lacZ*	This study
PAO1084	Gm^r^, 8485::mini-Tn*7*T-Gm-*mexE_p_-lacZ*	This study

Abbreviations: Cp, ciprofloxacin; Gm, gentamicin; *mexE_p_*, *mexE* promoter; ^r^, resistant; Tc, tetracycline; UWGC, University of Washington Genome Center.

To assess whether the increased fitness of PAO573 was due to expression of an efflux pump, we assessed the antibiotic susceptibility profile of this strain. PAO573 was highly resistant to chloramphenicol (MIC >1024 µg/mL) and trimethoprim (MIC >1024 µg/mL) when compared to PAO386 (in this strain both chloramphenicol and trimethoprim MICs were 16 µg/mL). Since both of these antibiotics are MexEF-OprN pump substrates [17] we suspected possible expression of this efflux system in PAO573.

To assess this notion, a ciprofloxacin resistant derivative was isolated by plating PAO386 onto LB agar supplemented with 0.05 µg/mL of ciprofloxacin, a condition known to select for MexEF-OprC expressing NfxC-type mutants. One colony growing after incubation at 37°C for 48 h was selected and the strain designated as PAO393. This mutant was also highly resistant to chloramphenicol and trimethoprim, exhibiting the same MIC values as PAO573 (>1024 µg/mL).

### Confirmation of MexEF-OprN expression in PAO393 and PAO573

**Figure 2:**
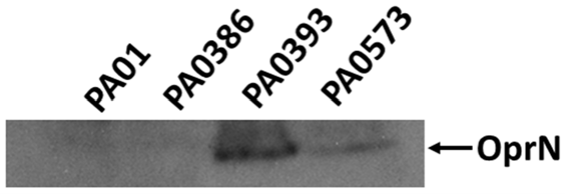
Immunodetection of OprN. Western blot analyses was performed on whole cell lysates of strains PAO1 (wild-type), PAO386 (PAO1 with Δ(*mexAB-oprM*) Δ(*mexCD-oprJ*) Δ(*mexJK*) Δ(*mexXY*) Δ*opmH mexT* with 8 bp insertion), PAO393 (ciprofloxacin resistant variant of PAO386 expressing MexEF-OprN *mexT^+^*), and PAO573 (LB-selected variant of PAO386 expressing MexEF-OprN *mexT* with 8 bp insertion) using anti-OprN antibodies to confirm the expression of OprN (indicated by the arrow). OprN was only detectable in extracts of PAO393 and PAO573.

To confirm MexEF-OprN expression in PAO393 and PAO573, we first performed Western blot analysis of whole cell lysates using anti-OprN antibodies (Fig. 2). OprN could not be detected in the parent strain PAO386 and PAO1, but it was expressed in PAO393 and PAO573.

**Figure 3:**
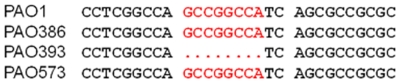
Expression of *mexEF-oprN* in *P. aeruginosa* strains as determined by *mexE*_promoter_-*lacZ* reporter fusion assays. β-galactosidase assays were performed on *P. aeruginosa* strains harboring single-copy *mexE*_promoter_-*lacZ* fusions on mini-Tn*7* elements integrated in the chromosome at the *att*Tn*7* site. PAO604, PAO1:: *mexE*_promoter_-*lacZ*; PAO605, PAO386:: *mexE*_promoter_-*lacZ*; PAO606, PAO393:: *mexE*_promoter_-*lacZ*; and PAO607,PAO573:: *mexE*_promoter_-*lacZ*. For relevant host strain genotypes see Fig. 2 legend.

Confirmation of the overexpression of the *mexEF-oprN* operon in the various mutant strains was also achieved by assessing *mexEF-oprN* transcription using transcriptional *mexE*_promoter_-*lacZ* fusions integrated in single-copy into the chromosomes of the respective mutants. PAO604 (PAO1::*mexE*_promoter_-*lacZ*) and PAO605 (PAO386::*mexE*_promoter_-*lacZ*) did not show any significant β-galactosidase activity (Fig. 3). In contrast, PAO606 (PAO393::*mexE*_promoter_-*lacZ*) and PAO607 (PAO573:: *mexE*_promoter_-*lacZ*) both showed significant *mexE*_promoter_-*lacZ* transcription. PAO607 expressed about seven-times more β-galactosidase activity than PAO606. DNA sequence analyses revealed that *mexEF-oprN* operon overexpression in PAO573 was not due to changes in the promoter region.

### MexEF-OprN expression in PAO573 is independent of MexT

**Figure 4:**
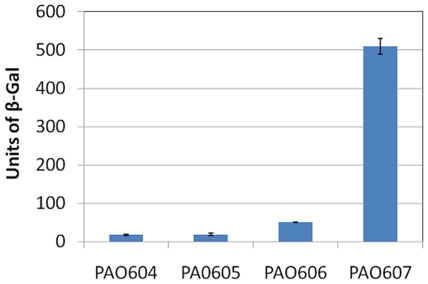
Confirmation of the presence of 8-bp insertion in the *mexT* gene. The *mexT* gene was PCR amplified from genomic DNA templates of PAO1, PAO386, PAO393 and PAO573, and sequenced to confirm the presence or absence of the 8-bp insertion (shown in red) after nucleotide 231. For relevant strain genotypes see Fig. 2 legend.

Since *lacZ* fusion and Western blot experiments confirmed transcriptional and translational expression of MexEF-OprN, we next probed the status of *mexT* in the two mutant strains since the parental strain PAO393 was derived from a PAO1 strain with an 8-bp insertion in *mexT*. PCR amplification and sequencing of the *mexT* gene from PAO1, PAO393 and PAO573 revealed that the 8-bp insertion was lost from PAO393 (the mutant isolated from ciprofloxacin-supplemented media) but not from PAO573 (the mutant strain isolated as a large colony variant on LB agar) (Fig. 4). This indicates MexT-dependent MexEF-OprN expression in PA393 but MexT-independent expression of this efflux pump in PAO573.

### Attempts at identification of *mexEF-oprN* regulatory proteins other than MexT

Since the *mexEF-oprN* operon expression was constitutively expressed in a MexT-independent manner in PAO573 we hypothesized that additional regulatory protein(s) may be involved in regulation of its expression. We attempted two experimental approaches to test this hypothesis, random mutagenesis of *mexE*_promoter_-*lacZ* bearing strains and identification of clones in a *P. aeruginosa* expression library that either up- or down-regulate β-galactosidase expression *in mexE*_promoter_-*lacZ* bearing strains.

### Random mutagenesis approaches

In order to identify elements involved in the regulation of MexEF-OprN pump, our first approach involved random mutagenesis using the *Mariner* transposon delivery vector pBT20 [34], of PAO604 (PAO1::*mexE*_promoter_-*lacZ*) in order to identify potential repressor protein(s). Since colonies of PAO604 are white on LB agar plates supplemented with X-gal, we screened for blue colonies following the transposon mutagenesis as an insertion in a repressor gene should result in increased expression of the β-galactosidase gene under the control of *mexE* promoter. However, in spite of several attempts, we were unable to find any mutants that showed up-regulation of β-galactosidase expression.

In an attempt to find potential activator(s), we attempted random mutagenesis screen in PAO607 (PAO573::*mexE*_promoter_-*lacZ* fusion). We assumed that if the expression of *mexEF-oprN* in this strain was due to an altered activity of an activator molecule then disruption of such molecule would result in white colonies on LB-agar supplemented with X-gal. However, our attempts repeatedly identified insertions in the *lacZ* gene only.

### Screening of a *P. aeruginosa* expression library

**Figure 5:**
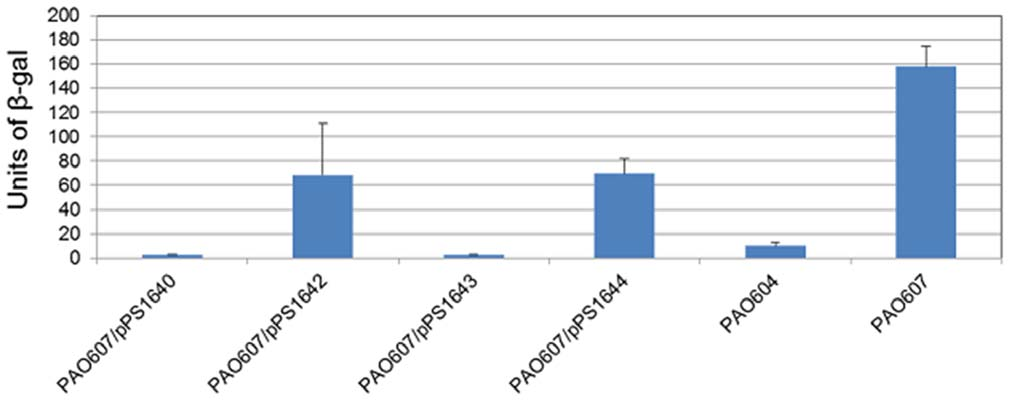
Screening of *P. aeruginosa* PAO1 library clones for repression of *mexEF-oprN* operon transcription. β-galactosidase assays were performed using the indicated *P. aeruginosa* strains with or without plasmids containing various PA genes. PAO604 is PAO1:: *mexE*_promoter_-*lacZ* (negative expression control) and PAO607 is PAO573:: *mexE*_promoter_-*lacZ* (positive expression control). Plasmids carry the following genes: pPS1640 (*PA2050* [probable sigma factor]); pPS1642 (partial sequence of *PA4315* [*mvaT*]); pPS1643 (*PA0486-PA0487* [molybdenum transport regulator]); and pPS1644 (*PA2489* [AraC-type regulator]-*PA2490* [hypothetical protein]-*PA2491* [*mexS*]-*PA2492* [*mexT*]).

Since transposon mutagenesis did not identify any regulatory protein candidates, we decided to search for a potential repressor using an alternative approach. We transformed PAO607 (PAO573::*mexE*_promoter_-*lacZ*) with a PAO1 plasmid library and identified four colonies harboring recombinant plasmids that could repress the expression of a β-galactosidase gene under the control of the *mexE* promoter (Fig. 5). Sequencing of the four recombinant plasmids thus identified – pPS1640, pPS1642, pPS1643 and pPS1644 - revealed several possible regulatory protein candidates.

**Figure 6:**
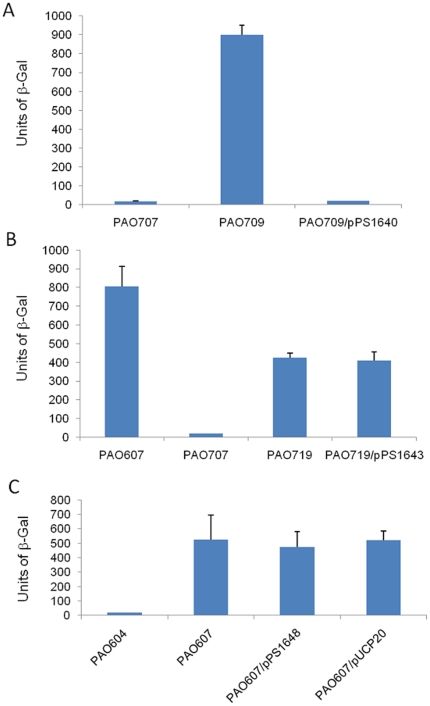
Effect of *PA2050*, *PA0487*, and *PA2489* on the expression of *mexEF-oprN* operon. **A. Deletion of** ***PA2050*** **in PAO707.** PAO709 contains a gene deletion of *PA2050* introduced into PAO707 which is PAO1 with Δ(*mexAB-oprM*) Δ(*mexCD-oprJ*) Δ(*mexXY*) and chromosomally-integrated *mexE*_promoter_-*lacZ*; pPS1640 contains the gene *PA2050* cloned in pUCP20. **B. Deletion of** ***PA0487*** **in PAO707.** PAO719 contains a gene deletion of *PA0487* in PAO707; pPS1643 contains the gene *PA0487* in pUCP20. **C. Expression of PA2489.** pPS1648 containing the *PA2489* gene was electroporated into PAO607 (PAO573::*mexE*_promoter_-*lacZ*); pUCP20 was used as the vector control. PAO604 is the PAO1:: *mexE*_promoter_-*lacZ* negative control and PAO607 is the PAO573::*mexE*_promoter_-*lacZ* positive control.

Plasmid pPS1640 harbored *PA2050* that encodes for a probable sigma factor. A recent study has speculated on the role of a yet unidentified sigma-factor in the expression of MexEF-OprN pump [35]. In order to avoid any inadvertent expression of the MexEF-OprN system in PAO386, we decided to use PAO327 for the follow up studies. This strain is a stable Δ(*mexAB-oprM*) Δ(*mexCD-oprJ*) Δ(*mexXY*) derivative of PAO1 and its *mexEF-oprN* operon is uninducible as a result of the 8-bp *mexT* insertion. We deleted *PA2050* from PAO707 (PAO327:*mexE*_promoter_-*lacZ*) to obtain PAO709, and observed a dramatic increase in β-galactosidase activity which could be repressed by the introduction of the plasmid-borne *PA2050* gene (Fig. 6A). However, the phenotype observed upon deletion of *PA2050* was not reproducible every time as transfer of the deletion back into PAO707 did not result in a consistent phenotype, nor did its deletion have any effect on the expression of MexEF-OprN pump in the wild type strain *P. aeruginosa* PAO1. Additionally, we did not find any mutations in the *PA2050* gene in PAO573 (data not shown).

Plasmid pPS1642 was also shown to repress the β-galactosidase activity in our reporter strain. This plasmid contains a 1,710 bp insert that includes a partial sequence of the *mvaT* gene, missing 124 bp of the 5′-end. Even though the insert contained only a partial sequence of the *mvaT* gene, the product of which has been implicated in the expression of *mexEF-oprN* operon [29], we used a *mvaT* insertion mutant available from the University of Washington Genome Center in order to investigate its role in the expression of MexEF-OprN system in our strain. We inserted the mini-Tn*7*T-*mexE*_promoter_-*lacZ* reported fusion in this strain (PAO1084), however the strain did not show any increased promoter activity compared to the derivative (PAO1081) containing the promoter-less *lacZ* insertion (data not shown).

The plasmid pPS1643 contains the ORFs *PA0486* and *PA0487*. *PA0486* encodes a protein with homology to serine/threonine kinases and *PA0487* encodes a probable molybdenum transport regulator. We decided to investigate its role in the expression of the *mexEF-oprN* operon. For reasons detailed above, we used again the PAO327 background and created PAO719 by deleting *PA0487* from PAO707 which is an unmarked derivative of PAO327 containing the *mexE*_promoter_-*lacZ* fusion. This strain not only exhibited increased β-galactosidase activity (Fig. 6B) but also elevated MICs for chloramphenicol and trimethoprim (512 µg/mL and >1024 µg/mL, respectively) when compared to the PAO719 parental strain which exhibited MICs for chloramphenicol and trimethoprim of 2 µg/mL and 32 µg/mL, respectively. However, introduction of pPS1643 (containing *PA0487*) gene did not cause a reduction in β-galactosidase activity (Fig. 6B). We created a number of mutants of PAO327 containing gene deletions of *PA0487*. However, as in the case of *PA2050* discussed above, the phenotype observed upon deletion of *PA0487* was not reproducible every time as the transfer of the deletion in PAO707 did not result in a consistent phenotype, nor did the deletion have any effect on the expression of MexEF-OprN pump in the wild-type PAO1. Additionally we did not find any mutations in the *PA0487* gene in PAO573 (data not shown).

Lastly, pPS1644 contains an approximately 4.5 Kb insert that includes the upstream region of *mexEF-opN* operon consisting of the *mexT* and *mexS* genes along with *PA2489*, a gene that encodes for a AraC family transcriptional regulator, and *PA2490* encoding a hypothetical protein. Since MexS was previously shown to be involved in the regulation of *mexEF-oprN* operon expression [27], we sequenced the *mexS* genes from PAO386 and PAO573. Sequence comparisons did not reveal any mutations in the *mexS* gene of PAO573 (data not shown). Also, since MexS activity is MexT-dependent and since *mexT* gene product in PAO573 remains inactive as a result of the 8-bp insertion, we do not believe that MexS plays a role in the repression of *mexE*_promoter_-*lacZ* encoded β-galactosidase activity by pPS1644. In order to investigate the possible role of *PA2489* (encoding an AraC family transcriptional regulator), we deleted a 2,291 fragment that contained *mexT* and 180 bp of the 3′-end of the *PA2490* gene thus effectively removing all three (*mexT*, *mexS*, and *PA2490*) genes from pPS1644 but leaving *PA2489* intact. However, transformation of the resulting pPS1648 into PAO607 did not result in any decrease in β-galactosidase activity (Fig. 6C).

## Discussion

In this study, we attempted to identify factors that control the expression of MexEF-OprN pump in the absence of antibiotic substrates. To avoid interference by other efflux systems we employed a *P. aeruginosa* strain that is lacking four different RND pumps (MexAB-OprM, MexCD-OprJ, MexJK, and MexXY). In addition, the strain also lacks the outer membrane protein OpmH, a TolC homolog, that has been shown to function with at least two different RND pumps in *P. aeruginosa*, MexJK [36] and TriABC [23]. We were readily able to isolate MexEF-OprN overexpressing mutants of this strain in the presence of media supplemented with ciprofloxacin. These mutants consistently showed a loss of the 8-bp insertion in the *mexT* gene resulting in expression of active MexT which induces the expression of the MexEF-OprN pump. However, in absence of any antibiotics in the media, colonies of PAO386 appear very small and have a pin-point morphology, some of which upon longer incubation give rise to a large colony variant. These large colony variants showed an increased expression of MexEF-OprN pump as confirmed in the representative strain PAO573 by increased MICs to MexEF-OprN substrates, detection of OprN by immunoblotting with anti-OprN antibodies (Fig. 2), β-galactosidase assays with strains expressing *mexE*_promoter_-*lacZ* transcriptional fusions (Fig. 3), and also qRT-PCR using *mexEF-oprN* operon specific primers (data not shown). Since the 8-bp insertion was still present in the *mexT* gene of PAO573, the overexpression of the MexEF-OprN operon was independent of MexT protein suggesting a role of other regulator(s) in the expression of this pump.

A search for such regulator(s) using a transposon mutagenesis approach was unsuccessful, but screening of a PAO1 expression library for clones capable of repressing β-galactosidase expression in a PAO573::*mexE*_promoter_-*lacZ* host revealed several candidates. We identified and sequenced four different recombinant plasmids from the screening of the PAO1 library, namely pPS1640 (containing the ORF *PA2050*), pPS1642 (containing the partial sequence of *PA4315*), pPS1643 (containing the ORFs *PA0486-PA0487*), and pPS1644 (containing *PA2489-mexS-mexT*), that were found to repress the expression of the reporter β-galactosidase gene in the host strain (Fig. 5). *PA0487* encodes a probable molybdenum transport regulator, *PA2050* encodes a probable sigma factor, while *PA4315* encodes for the transcriptional regulator MvaT. The insert in pPS1644 contains a gene (*PA2489*) that encodes an AraC family transcriptional regulator, a gene (*PA2490*) that encodes for a hypothetical protein, and also the *mexS* and *mexT* genes. While these genes repress *mexE* promoter activity in the PAO573 background, deletion and re-transformation analyses gave inconsistent results, especially *PA0487* and *PA2050*, and did not yield the same phenotypes, i.e. *mexEF-oprN* expression, when the respective genes were mutated in PAO1. Furthermore, when PCR-amplified from PAO573 the genes contained on and presumably expressed by the respective plasmids did not contain any mutations.

One of the reasons for inconsistent observations for deletion phenotypes for *PA0487* and *PA2050* could be the unstable nature of our mutants. One notable point is that the phenotype that we observed in PAO573 results from the deletion of OpmH, which may indicate that the possible physiological stress on this strain could be a cumulative effect of hampered activity of other yet uncharacterized pumps that may require OpmH for function, and the *mexEF-oprN* over-expressing phenotype is likely to be a result of accumulation of metabolic by-products or secondary metabolites. Also, our study is difficult to repeat in the wild-type strain since the activity of other pumps (that were deleted in PAO386) would most likely prevent the over-expression of MexEF-OprN pump.

In conclusion, our study shows that the expression of MexEF-OprN pump can be derepressed in absence of antibiotic stress as shown by some of the previous studies [32], [33]. We also provide further evidence for regulation of the expression of *mexEF-oprN* pump being under control of a very complex regulatory network and that additional studies are required to understand the underlying mechanisms. In support of this notion, a recent study postulated transcriptional regulation of the *mexEF-oprN* multidrug efflux operon by an unidentified repressor [34] but it remains pure speculation whether inactivation of this repressor might be the root cause for the observed *mexEF-oprN* operon over-expression in PAO573. In this context, however, it is interesting to note that just as deletion of the putative repressor-binding site caused high-level *mexEF-oprN* expression [34], the unknown mutation(s) present in PAO573 also caused a level of MexT-independent *mexEF-oprN* expression that was significantly higher than that observed in PAO393 where *mexEF-oprN* expression was MexT-dependent (Fig. 3). The most striking discovery of the present study is that antibiotic and metabolic stressors lead to MexEF-OprN over-expression by independent mechanism(s). Antibiotic stress caused *mexEF-oprN* over-expression via a MexT dependent mechanism whereas metabolic stress caused expression of this operon via a MexT-independent mechanism. While the nature of the metabolic signal(s) causing MexEF-OprN expression remain unknown, our results provide further evidence that RND efflux pumps not only extrude antimicrobials but rather serve other important metabolic functions.

## Materials and Methods

### Bacterial strains, plasmids and growth conditions

*P. aeruginosa* strains used in this study are listed in Table 1. *E. coli* DH5α (Invitrogen, Carlsbad, CA) was used for the gene cloning experiments. LB medium (EM Sciences, Gibbstown, NJ) was used for the growth of *E. coli* and *P. aeruginosa* strains. When required, the growth medium was supplemented with the following antibiotics: ampicillin (Ap) (Sigma, St. Louis, MO) (100 µg/mL, *E. coli*), carbenicillin (Cb) (Gemini Bioproducts, Sacramento, CA) (200 µg/mL, *P. aeruginosa*), and gentamicin (Gm) (Sigma) (30 µg/mL, *P. aeruginosa*).

MexEF-OprN overexpressing strains were isolated by streaking PAO386 on LB agar or LB agar supplemented with 0.05 µg/mL of ciprofloxacin (Cp; Sigma). A list of plasmids used in this study is provided in Table S1.

### Antibiotic susceptibility assays

Antibiotic susceptibility testing was performed for chloramphenicol and trimethoprim using the two-fold microdilution method as specified by the Clinical Laboratory Standards Institute [37]. Both antibiotics were purchased from Sigma.

### Polymerase chain Reactions, DNA manipulations, and genetic techniques

PCR reactions were performed using either *Taq* DNA polymerase (New England Biolabs, Beverly, MA) or HiFi high-fidelity *Taq* polymerase (Invitrogen, Carlsbad, CA).

Extraction of plasmid and genomic DNA and gel purification of the DNA was performed using kits available from Qiagen (Qiagen, Valencia, CA). Transfer of DNA into *P. aeruginosa* strains was achieved either by using tri-parental mating [38] or by the 10-minute rapid electroporation [39] methods described previously.

Construction of the reporter fusion using the promoter region of the *mexEF-oprN* operon (*mexE*_promoter_) and the promoter-less *E. coli lacZ* gene was accomplished as follows. The promoter region of the *mexEF-oprN* operon was amplified on a 683-bp fragment from PAO1 genomic DNA using *Taq* polymerase, primers 557 (5′-GCCAGCTGCAGCTCGACGACTATTGCG; a *Pst*I site is underlined) and 558 (5′-TTTCCGGAAGCTTGCCGCAGGCGCTCA; a *Hin*dIII site is underlined), and cloned into the TA cloning vector pCR2.1 (Invitrogen, Carlsbad, CA) to obtain pPS1496. Next, a 420-bp DNA fragment containing the 36 carboxy-terminal *mexT* codons, the 29 amino-terminal *mexE* codons and the 230-bp *mexT-mexE* intergenic region with the *mexE* promoter was obtained after digestion of pPS1496 DNA with *Hin*dIII and *Sma*I. The *Sma*I-*Hin*dIII fragment was gel-purified, and cloned into the reporter plasmid pPS1453 (pUC18-mini-Tn*7*T-Gm-*lacZ*) [40] digested with the same enzymes to derive pPS1519. Assembly of the *mexE*_promoter_-*lacZ* fusion construct in a mini-Tn*7* vector allows the insertion of the reporter fusion in single copy in the *P. aeruginosa* chromosome, which was achieved using the helper plasmid pTNS2 [40] and a previously published protocol [40]. Confirmation of the insertion and the subsequent removal of the Gm^r^-cassette was performed by protocols described previously [40].

Creation of unmarked gene deletions in *P. aeruginosa* was performed using previously described methods [41].

### Immunodetection of OprN

Western blot detection of the OprN protein (the outer membrane component of the MexEF-OprN pump) was performed using an anti-OprN polyclonal antiserum. One mL of bacterial cells grown in LB broth to a density of A_600_ ∼1.0 were harvested and normalized using 2× sample buffer (0.0025 w/v bromphenol blue, 20% v/v glycerol, 6% v/v β-mercaptoethanol, 2.5% w/v SDS in Tris Buffer). Normalization was carried out as follows, A_600_×0.08 = µL 2× sample buffer. The cell suspension was boiled for 5 minutes and an equal volume of each sample (3–5 µL) was loaded on a 10% SDS-polyacrylamide gel. Immunodetection of the OprN protein was carried out using previously described protocols [42].

### β-galactosidase assays

Reporter strains containing the *mexE*_promoter_-*lacZ* fusion inserted in the genome were constructed as described above for PAO1, PAO386, PAO393, and PAO573 to derive PAO604, PAO605, PAO606, and PAO607, respectively.

β-galactosidase assays were performed using a previously described method [43]. Briefly, overnight cultures of bacterial strains grown in LB-broth at 37°C were subcultured in fresh LB using a 1∶200 inoculum and cultures were grown until they reached an optical density of approximately one at 600 nm (A_600_ = 1.0), at which point, one mL aliquots were removed and cells harvested at 13,000 rpm in a microcentrifuge. Cell pellets were resuspended in 0.1 M phosphate buffer (pH 7.0). Permeabilization of cells was achieved by addition of SDS/chloroform. Assays of β-galactosidase activity and activity unit calculations were performed as described previously [44].

### Construction of *P. aeruginosa* library

A *P. aeruginosa* library was constructed by partially digesting genomic DNA from the wild-type PAO1 with *Eco*RI and *Eco*RI+*Bam*HI and purifying the DNA fragments ranging from approximately 1.5 Kb to 4 Kb from an agarose gel. The DNA fragments were dephosphorylated using alkaline phosphatase (New England Biolabs) following the manufacturer's instructions and then ligated into the broad-host range cloning and expression vector pUCP20 [45] digested with *Eco*RI and *Eco*RI+*Bam*HI, respectively. The ligation mixtures were transformed into *E. coli* DH5α cells and plated on LB agar supplemented with ampicillin (100 µg/mL) and X-gal (5-bromo-4-chloro-3-indolyl-β-D-galactopyranoside; 40 µg/mL) (Gold Biotechnology, St. Louis, MO). Following an overnight incubation at 37°C, 825 white colonies were selected and patched on fresh LB agar plates supplemented with ampicillin (100 µg/mL) and X-gal (40 µg/mL). The patches were washed off using saline and inoculated into LB broth supplemented with ampicillin, plasmids were extracted following an overnight incubation at 37°C with shaking, and electroporated into PAO607 (the derivative of PAO573 containing the chromosomally-integrated mini-Tn*7*T-*mexE*_promoter_-*lacZ* fusion). Cells were plated on LB agar supplemented with Cb (200 µg/mL) and X-gal (40 µg/mL). Following an overnight incubation at 37°C, four white to light blue colonies were observed and selected for further analysis. Plasmids were extracted from these four *P. aeruginosa* PAO607 transformants and sequenced at Colorado State University's Proteomics and Metabolomics Facility.

## Supporting Information

Table S1 List of plasmids used in this study.
(DOCX)
