# Supplementary material for: Evidence of MexT-Independent Overexpression of MexEF-OprN Multidrug Efflux Pump of Pseudomonas aeruginosa in Presence of Metabolic Stress
Source: PLoS One. 2011 Oct 24;6(10):e26520. doi: 10.1371/journal.pone.0026520 (PMC3200333; doi:10.1371/journal.pone.0026520)
Supplement: Table S1 — List of plasmids used in this study. (DOCX) [file pone.0026520.s001.docx]

**Table S1. List of plasmids used in this study**

| **Plasmid** | **Relevant characteristics** | **Source/Reference** |
| --- | --- | --- |
| pCR2.1 | Ap^r^; PCR cloning vector | Invitrogen |
| pTNS2 | Ap^r^; helper plasmid encoding the site-specific TnsABCD Tn*7* transposition pathway | [[1](#_ENREF_1)] |
| pFLP2 | Ap^r^; source of *Flp* recombinase | [[2](#_ENREF_2)] |
| pUCP20 | Ap^r^; broad-host-range cloning vector. | [[3](#_ENREF_3)] |
| pPS1453 | Ap^r^ Gm^r^; pUC18T-mini-Tn*7*T-Gm-*lacZ*; mini-Tn*7* vector containing the promoter-less *lacZ* gene | [[4](#_ENREF_4)] |
| pPS1496 | Ap^r^; pCR2.1 containing the PCR-amplified *mexE*_promoter_ region on a 683-bp fragment*.* | This study |
| pPS1519 | Ap^r^ Gm^r^; pPS1453 containing the *mexE*_promoter_ on a 420-bp fragment | This study |
| pPS1640 | Ap^r^; pUCP20 containing a 3,011 bp *EcoR*I-*Bam*HI fragment carrying the *PA2050* gene PAO1. The insert also contains partial sequences of *PA2049* and *PA2051*. | This study |
| pPS1642 | Ap^r^; pUCP20 containing a 1,710 bp *Eco*RI-*Bam*HI fragment containing the partial sequences of *PA4315* (*mvaT*)(missing 124 bp from the 5’-end) and *PA4316* (*sbcB*) (missing 67 bp from the 5’-end) | This study |
| pPS1643 | Ap^r^; pUCP20 containing a 2,317 bp *Eco*RI-*Bam*HI fragment containing *PA0486* (protein with homology to serine/threonine kinases) and *PA0487* (molybdenum transport regulator). | This study |
| pPS1644 | Ap^r^; pUCP20 containing a 4,494 bp *Eco*RI-*Bam*HI fragment containing partial sequence of *PA2488* (transcriptional regulator of AraC family) (missing 157 bp from 5’-end), *PA2489* (transcriptional regulator of AraC family), *PA2490* (hypothetical protein)*, PA2491* (*mexS*), *PA2492* (*mexT*), and the partial sequence of *PA2493* (*mexE*) (containing 1,244 bp of 5’-end) | This study |
| pPS1648 | Ap^r^, pPS1644 without the 2,291 *Cla*I-*Sma*I fragment that contains the *mexT* and *mexS* genes and 120 bp of 3’-end of *PA2490* gene. The plasmid thus contains only the *PA2489* gene. | This study |

Abbreviations: Ap, ampicillin; Gm, gentamicin; ^r^, resistant

**References**

1. Choi KH, Gaynor JB, White KG, Lopez C, Bosio CM, et al. (2005) A Tn*7*-based broad-range bacterial cloning and expression system. Nat Methods 2: 443-448.

2. Hoang TT, Karkhoff-Schweizer RR, Kutchma AJ, Schweizer HP (1998) A broad-host-range *Flp*-FRT recombination system for site-specific excision of chromosomally-located DNA sequences: application for isolation of unmarked *Pseudomonas aeruginosa* mutants. Gene 212: 77-86.

3. West SEH, Schweizer HP, Dall C, Sample AK, Runyen-Janecky LJ (1994) Construction of improved *Escherichia-Pseudomonas* shuttle vectors derived from pUC18/19 and the sequence of the region required for their replication in *Pseudomonas aeruginosa*. Gene 128: 81-86.

4. Choi KH, Schweizer HP (2006) mini-Tn*7* insertion in bacteria with single *att*Tn*7* sites: example *Pseudomonas aeruginosa*. Nat Protoc 1: 153-161.
